# Floral Volatile Organic Compounds of *Mitchella repens* (Rubiaceae)

**DOI:** 10.1002/pei3.70022

**Published:** 2024-12-15

**Authors:** Aleel K. Grennan, Kathleen C. Murphy, Mary Fowler, Adam Bengtson, Jay Turner, Lucas Horan, Julia Fitzpatrick, Logan Desilets

**Affiliations:** ^1^ Biology Department Worcester State University Worcester Massachusetts USA; ^2^ Chemistry Department Worcester State University Worcester Massachusetts USA; ^3^ Mathematics Department Worcester State University Worcester Massachusetts USA

**Keywords:** distyly, floral VOCs, GC–MS, *Mitchella repens*, SPME

## Abstract

*Mitchella repens* (partridgeberry; family Rubiaceae) is a creeping, understory plant native to eastern North America. The twinned, tubular flowers of this distylous plant are bright white and produce volatile organic compounds (VOCs). Partridgeberry has intermorph incompatibility and thus requires pollinators to move pollen from one morph to the other. Despite partridgeberry being a common member of forest communities, little is known about its pollination syndrome. Using headspace solid‐phase microextraction (HS‐SPME) coupled with gas chromatography–mass spectrometry (GC–MS) analysis the floral VOCs were identified, with the four predominant molecules being α‐pinene, camphene, D‐limonene, and verbenone. The VOC profile contained 27 molecules consisting mostly of monoterpenes. Two independent sample *t*‐tests confirmed that each morph produced statistically similar floral VOC profiles (*p* > 0.1). Additionally, two of the predominant VOC molecules, α‐pinene and D‐limonene, were measured throughout the 5‐day flowering cycle. Simple linear regressions of these compound levels versus days after flowering (DAF) confirmed that α‐pinene and D‐limonene both decreased with flower age. Insect visits were observed to correlate with α‐pinene and D‐limonene concentrations, peaking at 1–2 DAF and then declining through 5 DAF.

## 1 Introduction

**FIGURE 1:**
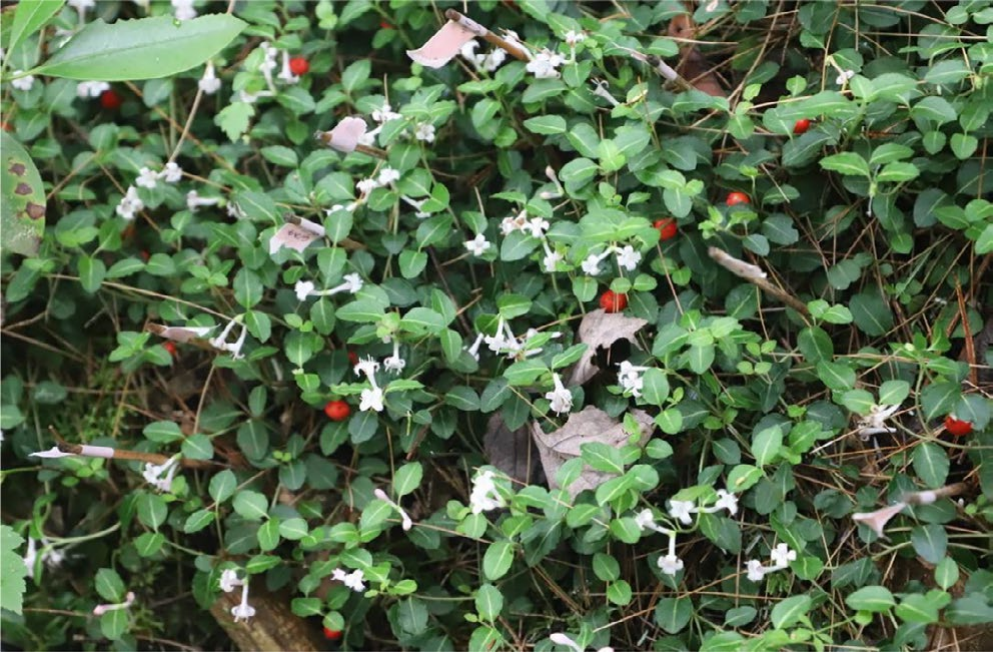
Partridgeberry mat from the study location in Holden, MA. The twinned flowers and red berries from last season are clearly seen. Flags are marking the floral age (days after flowering [DAF]).

*Mitchella repens* , commonly known as partridgeberry, is a perennial dicot endemic to the eastern half of the United States and Canada. It is one of the two *Mitchella* species found in the family Rubiaceae, the other *Mitchella undulata*, has a similar morphology and ecology but it is native to Japan and Korea. Partridgeberry thrives in shaded undisturbed areas with a preference for dark, humus‐rich acidic soils. The creeping runners of the plants intertwine and spread throughout the forest floor creating a thick carpet of paired green leaves (Figure 1). The dense mat‐like growth of the plant provides shelter to numerous invertebrates. In late spring and early summer, the plant produces pairs of scented, white tubular flowers that share a common ovary with eight ovules (Keegan, Voss, and Bawa 1979; Hicks, Wyatt, and Meagher 1985).

Partridgeberry is a classic distylous plant, producing two floral morphs, pin and thrum, borne on separate plants (Keegan, Voss, and Bawa 1979; Barrett, Jesson, and Baker 2000; Barrett 2002). The pin has an elongated carpal surrounded by shorter stamens, while the thrum flowers have elongated anthers surrounded by a shortened carpal. The plant exhibits heteromorphic self‐incompatibility: fertilization of pin flowers requires pollen from a thrum and fertilization of thrum flowers requires pollen from a pin. Additionally, both paired flowers require successful pollination for fertilization and fruit formation to occur (Keegan, Voss, and Bawa 1979; Hicks, Wyatt, and Meagher 1985).

The requirement of intermorph pollination in partridgeberry highlights the importance of pollinator attraction for this species. Within a partridgeberry population, the distribution of pin and thrum flowers is not equal, suggesting the need for pollinators to fly longer distances (Hicks, Wyatt, and Meagher 1985). Previous studies have identified bumblebees ( *Bombus vagans* and *B. impatiens* ) as the main pollinator of partridgeberry (Hicks, Wyatt, and Meagher 1985). However, bumblebees are not always prevalent in the locals where partridgeberry grows, indicating that there might be other pollinators involved to support its reproduction.

To attract pollinators, flowers emit a scent to provide a “road map” (Muhlemann, Klempien, and Dudareva 2014; Mostafa et al. 2022). This scent or bouquet is a complex mixture, consisting of many volatile organic compounds (VOCs; Endress 2006; Muhlemann, Klempien, and Dudareva 2014; Hetherington‐Rauth and Ramírez 2016; Rebolleda‐Gómez et al. 2019). Within 90 plant families studied, approximately 1700 chemical compounds have been identified in floral scents (Knudsen et al. 2006; Muhlemann, Klempien, and Dudareva 2014). Generally, these volatile molecules are classified as terpenoids, benzenoids, and phenylpropanoids (Stashenko and Martínez 2008; Slavković and Bendahmane 2023), with the monoterpenes being the most common (Dunkel et al. 2009; Lo et al. 2021a, 2021b; Wang et al. 2021). The quantity and profile of the emitted floral VOCs have been shown to depend on floral age, position on the plant (i.e., distance from main stem), as well as fertilization status (Marshall et al. 2010; Burdon et al. 2015; Burkel and Runyon 2017). The types of VOCs emitted by partridgeberry have not yet been investigated. Additionally, it is uncertain if each morph produces a different VOC profile, as has been observed in other distylus species (Obi Johnson et al. 2019).

The most efficient method to identify flower VOCs is using headspace solid‐phase microextraction (HS‐SPME) followed by gas chromatography–mass spectrometry (GC–MS) analysis (Condurso et al. 2016; Silva et al. 2018; Kim et al. 2020; Lo et al. 2021a, 2021b, 2024; Wang et al. 2021). This is a fast and sensitive technique that avoids the use of cumbersome instrumentation and organic solvents (Koziel and Pawliszyn 2001). There are many choices in the chemical composition of the polymer coatings of SPME fibers and arrows (Agilent, USA). Each type has a different sensitivity for the polarity and volatility of the analytes (Koziel and Pawliszyn 2001). The optimal coating to use for identifying floral VOCs has been investigated extensively (Song et al. 2007; Silva et al. 2018; Kim et al. 2020; Lo et al. 2021a, 2021b; Wang et al. 2021). The carbon wide‐range/polydimethylsiloxane (CWR/PDMS) coating is most efficient in extracting VOCs with a wide range of molecular mass and volatility (Silva et al. 2018).

To better understand partridgeberry pollinator attraction, populations in Holden, MA, U.S. were studied for three seasons during their flowering cycle; this occurred for about 4 weeks mid‐June to mid‐July. The VOCs emitted from the flowers were measured and the VOCs from pins were compared to VOCs from thrums and the concentrations of two predominant VOC molecules were measured each day throughout the 5‐day flowering cycle. Additionally, the frequency of insect visits to the flowers was recorded.

## 2 Methods

### 2.1 Study Site Description

Our study focused on partridgeberry populations located along the Massachusetts Central Rail Trail in Holden, MA, USA (Worcester County; 42°22′26.8″ N 71°49′50.7″ W). The dominant tree found at the study site is *Tsuga canadensis* (Eastern hemlock) with *Viburnum acerifolium* (maple leaf viburnum) as the most common understory shrub. The study populations grew on a moderately sloped hill just above the trail. A constantly flowing streamlet is located between the trail and incline keeping soil consistently moist. The area has a typical Northeast climate with cold winters and lower precipitation (mean January temperature, 0/−8°C; 43 cm average precipitation) and warm summers with more precipitation (mean July temperature, 27/18°C; 98 cm average precipitation).

Five independent patches of plants, measuring around 1 m^2^ and at least 3 m from another patch, were selected; far enough apart to ensure they were not from the same clones but close enough to have the same environmental conditions (Figure 1). Most patches contained pin and thrum plants but were dominated by thrums (60%–70%). Once flower buds became visible, the patches were monitored daily to mark the onset of flowering. On the first day, a flower appeared it was marked as 1 day after flowering (1 DAF). When flowers were harvested for analysis, the DAF was recorded. Over time, the flower aged and fell off the plant by 6 DAF.

### 2.2 Insect Visitor Observations

Insect visitor observations were conducted in person during the first two summers (19 h in 2020 and 6.25 h in 2021) between the hours of 10:00 and 14:00 and were not done on rainy days. Observers remained at the site for the 4‐h observation window. The number and insect order were recorded as was the DAF. A “visit” was more than just landing on the flower; substantial interaction (more than 1 s) with at least one of the twinned flowers. These insect visitors were considered potential pollinators and their identification is part of a future study.

### 2.3 Collection of Flowers for VOC Analysis

Between 9:00 and 11:00 AM, twinflower pairs were clipped below the ovary, immediately placed in a 20‐mL headspace vial, and sealed with an 18‐mm magnetic screw cap containing PTFE/Si septa (Agilent, USA). The DAF, type of morph (pin or thrum), and patch ID were recorded. All samples were collected in the morning on days with no precipitation but there were natural variations in the weather of the previous days and evenings.

### 2.4 Identification of VOC Profile Molecules

Twelve flower samples, six pins and six thrums, all of 2 DAF, were collected to identify and compare VOCs in both morph types. The vials containing the flowers remained sealed at ambient temperature until delivered to the laboratory. A SPME arrow coated with CWR/PDMS (outer diameter 1.5 mm, phase thickness 120 μm, phase length 20 mm; Agilent, USA) was inserted through the septa of the headspace vial to adsorb emitted VOCs. To identify the complete aromatic profile of partridgeberry, containing both low and high volatile compounds, two extraction temperatures were used in tandem for each sample, high (60°C) and room temperature (28°C; Wang et al. 2021). First, the SPME was exposed in the headspace and then the system was placed in a 60°C oven for 30 min. Then, keeping the SPME in the headspace, the vial was removed from the oven and extraction continued for an additional 10 min at room temperature. After the combined 40‐min extraction period, the SPME arrow was retracted from the vial and immediately inserted into the inlet of the GC at 250°C for 3 min allowing for thermal desorption. VOCs were separated on a chromatographic column followed by election‐ionization mass spectrometry (EI‐MS) detection. The GC used was an Agilent 7820 A coupled with an Agilent 5977B MSD as the EI‐MS. Chromatographic separation was performed using HP‐5MS Ultra inert capillary column (30 m x 250 um x 0.25 um film thickness, Agilent, USA). Analytical conditions were as follows: Ultra Inert straight liner (2 mm ID, Agilent), splitless injection at 250°C; carrier gas of helium at a flow rate of 1 mL/min; GC column temperature 35°C for 5 min then ramp by 5°C/min to 240°C, and hold for 5 min, for a run time of 51 min. The EI‐MS detection had the following parameters: electron ionization at 70 eV, auxiliary transfer line set to 250°C, MS source at 230°C, MS quadrupole at 150, full‐scan acquisition with range of 50–550 m/z. VOCs were identified using mass spectra of the National Institute of Standards and Technology (NIST) library with a similarity above 85%. The majority peaks, classified as those appearing in at least two of the 12 samples, were auto‐integrated and their normalized percent areas were calculated (MassHunter Quantitative Analysis). The retention index (RI) was calculated for all compounds using an alkane mixture (Sigma Aldrich) run under identical GC–MS conditions of the method and then the RI was compared to literature values. Additionally, authentic standards (Sigma Aldrich) were used to further confirm retention times of α‐pinene, β‐pinene, D‐Limonene, and 3‐Carene. SPME Fibers were preconditioned per manufacturer instructions and blanks and postanalysis runs confirmed that there was no carry‐over of contaminant peaks.

### 2.5 VOCs Versus Day of Flowering

To observe VOC trends throughout the flowering cycle, two of the prominent molecules identified in the VOC profile, α‐pinene and D‐Limonene, were quantified in 95 flower samples harvested 1–5 DAF. The flower samples were collected as described above and DAF was recorded (Table 1). For this analysis, pins and thrums were collectively grouped.

**TABLE 1:** Number of pins and thrums harvested according to their days after flowering (DAF). The measured VOC values were used to determine the correlation between VOCs and DAF.

	DAF				
	1	2	3	4	5
Pin	7	11	6	5	5
Thrum	16	11	14	13	7

It was assumed that the 2‐ to 3‐h time lapse between sample collection in the field and laboratory analysis allowed the VOCs to reach headspace equilibrium and SPME extraction at room temperature began immediately upon returning to the laboratory. The α‐pinene and D‐Limonene were detectable using 10‐min room temperature SPME extraction, thus heating the vials in the oven was not necessary (Eckert et al. 2018; Wang et al. 2021). Additionally, because in this portion of the study, only two molecules of the entire bouquet were of interest and a large number of samples needed to be analyzed, the GC run time was shortened and modified as follows: GC column temperature program started at 60°C, held for 1 min, ramped to 80°C at 2.5°C/min, ramped to 110°C at 5°C/min, ramped to 250°C at 15°C/min, total run time of 24.3 min. The elution of molecules before and after α‐pinene and D‐limonene were the same when using the previously described VOC‐profile oven program and this shorter oven program; confirming that there are no coeluting peaks interfering when the oven program is reduced to 24.3 min.

### 2.6 Data and Statistical Analysis

#### 2.6.1 Characterization of VOCs From Pins and Thrums

Two independent sample *t*‐tests using R software were performed using normalized percent areas to determine if there are statistically significant differences in the relative amounts of compounds emitted from each morph. The normalized percent peak areas from the four common compounds found in six pins and six thrums were used: α‐pinene, camphene, D‐limonene, and verbenone.

#### 2.6.2 Analysis of VOCs Versus DAF

Peak areas of α‐pinene and D‐limonene of the data set (95 flowers) were graphed versus DAF. For each of the two compounds, a multiple linear regression was done to determine if the concentration of the compound was related to time. Initially, time and morph variables were included. Significant correlation between morph and VOC concentration was not found when using two independent sample *t*‐tests. Subsequently, simple linear regressions were used to determine the relationship between compound concentration and DAF.

## 3 Results

### 3.1 Insect Observations

**FIGURE 2:**
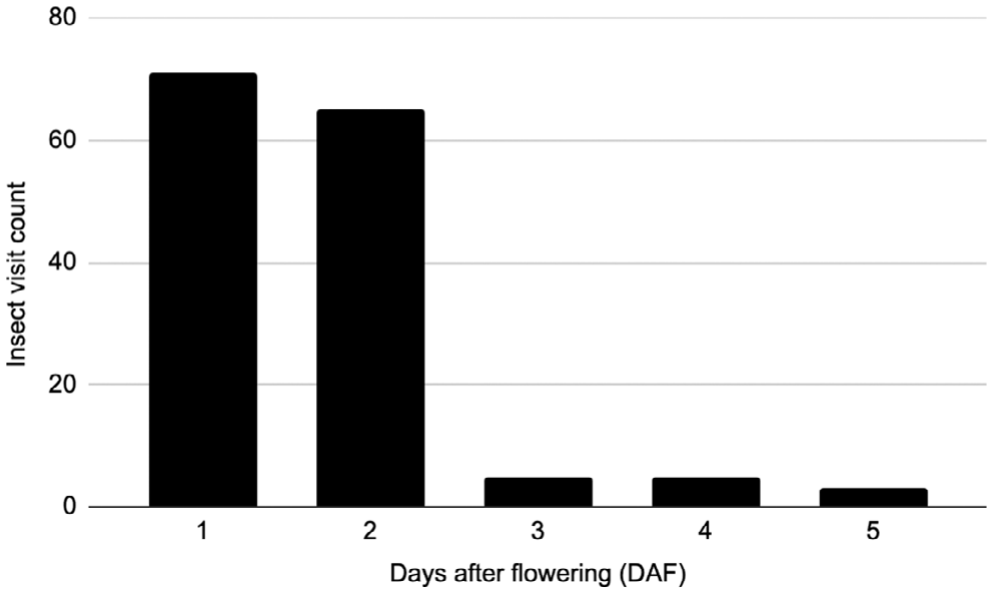
Insect visits based on days after flowering (DAF). All insect visitors with extended interaction with the flowers are included. Accidental visitors, such as ants and slugs, are not included.

Individual insect visits were scored according to the number of flowers they visited and floral age (DAF; Figure 2). An insect visit was the insect landing on a flower and staying longer than 1 s. The majority of visits were to younger flowers, 1 and 2 DAF, with the remaining DAF being significantly less.

### 3.2 Identification of VOC Profile

The VOCs identified in the mixed morph samples, six pins and six thrums, all of 2 DAF, are listed in Table 2. The majority peaks, classified as those appearing in at least two of the 12 samples, were integrated and their normalized percent areas (MassHunter Quantitative Analysis) and corresponding standard deviations were calculated. Area percentages of all compounds in the 12 flowers, sample chromatograms using raw MassHunter data, and NIST MS identification results are available in supplemental data. Four compounds were detected in all 12 samples: α‐pinene, camphene, D‐limonene, and verbenone, with α‐pinene and verbenone having the largest mean areas (Table 2).

**TABLE 2:** Partridgeberry VOC profile obtained from six pins and six thrums harvested 2 days after flowering.

	Family	Compound	Retention time (min)	Mean normalized area (%) (standard deviation)^a^		Retention index^b^	Identification^c^
				Pin (*n* = 6)	Thrum (*n* = 6)		
1	Monoterpene	α‐Thujene	10.47	0.17 (0.05)	0.16 (0.07)	927	MS + RI
2	Monoterpene	α‐Pinene	10.60	37 (8)	30 (10)	931	MS + RI + STD
3	Monoterpene	Camphene	11.12	1.6 (0.5)	1.4 (0.6)	947	MS + RI
4	Monoterpene	β‐Thujene	11.48	0.9 (0.4)	0.7 (0.5)	954	MS + RI
5	Monoterpene	β‐Pinene	12.21	1.2 (0.5)	1.2 (0.8)	974	MS + RI + STD
6	Monoterpene	β‐Myrcene	13.03	0.1	0.1 (0.2)	995	MS + RI
7	Monoterpene	4 (10)‐Thujene	13.10	0.3	0.2	997	MS + RI
8	Monoterpene	Bornylene	13.18	0.04	0.0	999	MS + RI
9	Monoterpene	1,3,8‐p‐Menthatriene	13.54	0.1	0.08	1010	MS + RI
10	Monoterpene	Α‐Phellandrene	13.66	0.04	0.1	1014	MS + RI
11	Monoterpene	α‐Terpinene	13.90	0.03	0.009	1021	MS + RI
12	Monoterpene	D‐Limonene	14.12	12 (6)	12 (6)	1027	MS + RI + STD
13	Monoterpene	γ‐Terpinene	15.32	0.1	0.2 (0.3)	1063	MS + RI
14	Monoterpene	3‐Carene	15.46	0.5	0.05	1067	MS + RI + STD
15	Monoterpene	Terpinolene	16.23	0.9 (0.1)	0.7 (0.6)	1089	MS + RI
16	Monoterpene	Borneol	18.83	2 (1)	1.8 (0.6)	1172	MS + RI
17	Monoterpene	Isoborneol	19.06	0.1	0.1	1180	MS + RI
18	Monoterpene	Verbenone	20.15	36 (9)	40 (20)	1216	MS + RI
19	Monoterpene	3‐Picoline	21.38	1 (2)	0.6	1259	MS + RI
20	Fatty acid derivative	Nonanal	17.11	0.1	0.4 (0.5)	1117	MS + RI
21	Fatty acid derivative	Isopropyl palmitate	38.95	0.1 (0.2)	0.0	2023	MS + RI
22	Formide	Formanilide	20.59	5	5	1231	MS + RI
23	Pyridine	2‐Picoline‐6‐carboxaldehyde	22.16	0.2	0.2 (0.5)	1286^d^	MS
24	Cycloheptane	2‐Aminotropone	22.29	0.4	1 (1)	1291^d^	MS
25	Benzaldehyde	p‐anisaldehyde	22.80	0.07	1	1309	MS + RI
26	Benzamides	DEET	30.78	0.09	0.7	1629	MS + RI
27	Hydrocarbon	Octadecane	43.72	0.03	0.02	1792	MS + RI

^a^ Standard deviation omitted for compounds found in less than three flower samples.

^b^ Calculated retention index (RI) using an HP 5MS column with temperature program.

^c^ MS = comparison to mass spectrum with NIST library, RI = comparison of calculated RI to literature RI value found online https://webbook.nist.gov/cgi/cbook.cgi?ID=002867‐05‐2&Units=SI&cGC=on#ref‐5, STD = comparison of mass spectra and retention times to an authentic standard.

^d^ No available literature RI values.

### 3.3 Pin VOCs Versus Thrum VOCs

The results of two independent sample *t*‐tests, based on normalized area percentages of the common four compounds (α‐pinene, camphene, D‐limonene, and verbenone) identified in all 12 samples (Table 2) concluded that there is no significant difference between pin VOCs and thrum VOCs (*p* > 0.1; Table 3).

**TABLE 3:** Calculated *t* and *p* values comparing the four common VOC molecules found in the floral morphs (six pin and six thrum flowers). Two independent sample *t*‐tests using R software were performed to investigate if there are statistically significant differences in the normalized percent areas of the four common compounds between the two floral morphs. Based on the calculated *p* values, no significant differences were found (*p* > 0.1). Normalized mean percent areas and standard deviations from Table 2 are included for reference.

Compound	*T*	*p*	Pin (*n* = 6)	Thrum (*n* = 6)
			Mean percent area (SD)	Mean percent area (SD)
α‐pinene	1.345	0.211	37 (8)	30 (10)
Camphene	0.480	0.643	1.6 (0.5)	1.4 (0.6)
D‐Limonene	0.128	0.901	12 (6)	12 (6)
Verbenone	−0.118	0.910	36 (9)	40 (20)

### 3.4 Do VOC Profiles Change With Floral Age?

**FIGURE 3:**
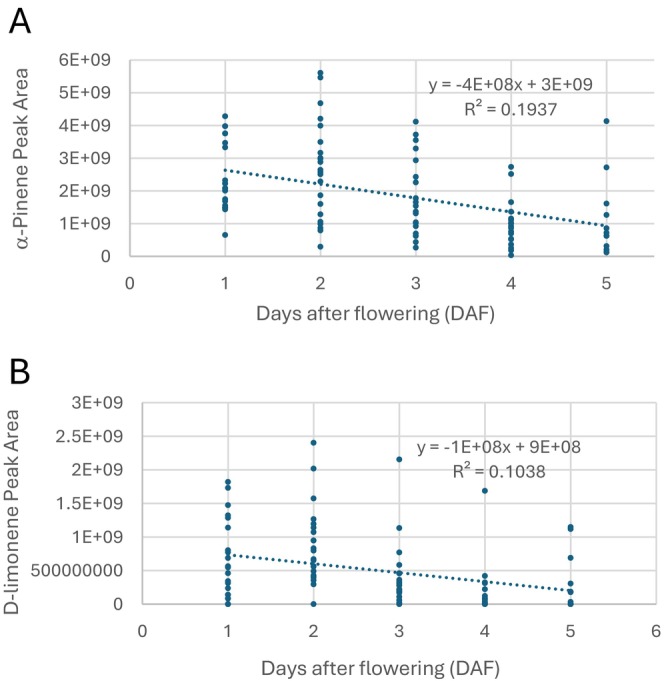
Peak areas versus days after flowering (DAF). (A) α‐Pinene. DAF is significant with *t* = −4.727 and *p* < 0.001 (B) D‐Limonene. DAF is significant with *t* = −3.282 and *p* = 0.001.

The peak areas of α‐pinene and D‐limonene measured throughout the 1–5 DAF are contained in Figure 3 (raw data available in supplemental data). For each of the two compounds, simple linear regression was used to determine the relationship between peak areas and DAF. Both of these compounds statistically changed with DAF; for α‐pinene, DAF is significant with *t* = −4.724 and a *p* < 0.0001 (Figure 3A) and for D‐limonene, DAF is also significant with a *t* = −3.282 a *p* = 0.001 (Figure 3B).

## 4 Discussion

As discussed previously, distylus plants, such as partridgeberry, have two floral morphs produced by genetically distinct plants; pins with long styles and short anthers and thrums with short styles and long anthers. Morph incompatibility is common in distylus plants, chemically preventing pins from pollinating pins and thrums from pollinating thrums (Charlesworth and Charlesworth 1979; Barrett 2002; Shao et al. 2019; Yuan et al. 2019; Lázaro, Seguí, and Santamaría 2020). At our study sites, patches of partridgeberries were typically dominated by one floral morph. The unique distylus physiology coupled with the isolation of plant morphs within patches would favor pollinators able to carry pollen long distances. The majority of insects observed visiting the flowers in our study were between 1 and 2 cm in length and dominated by bee and fly species. Identification of the insect visitors is part of a current study. It was also observed that the majority of insect visits were to younger flowers, peaking at 2 DAF and then decreasing. This decrease in pollinator visits correlated with a decrease in α‐pinene and D‐limonene concentrations (Figure 3) which suggests a possible causation.

Although the pollination syndrome of partridgeberry has been investigated previously (Keegan, Voss, and Bawa 1979; Hicks, Wyatt, and Meagher 1985), no studies have looked at the floral VOCs or reported additional pollinators. The chemical profile of partridgeberry floral VOCs was examined using six pins and six thrums, all DAF 2. As shown in Table 2, partridgeberry's VOC profile contains 27 unique compounds with the majority (19) as monoterpenes. The prevalent monoterpenes are α‐pinene, camphene, D‐limonene, and verbenone, with α‐pinene and verbenone contributing to the largest percent areas. Our results are consistent with the literature; monoterpenes are the most common floral VOC types found in the majority of plant families (Wang et al. 2021), and α‐pinene and D‐limonene are found in 67% and 71% of the families investigated to date, respectively (Knudsen et al. 2006).

The statistical analysis of pin and thrum VOCs confirmed that the two morphs emit a similar bouquet (*p* > 0.1; Table 3). Although some distylus species have been observed to have different VOC profiles (Obi Johnson et al. 2019), partridgeberry does not.

Floral VOCs have been shown to decrease as flowers age, possibly due to a decline in VOC production once flowers are fertilized (Marshall et al. 2010; Farré‐Armengol et al. 2020; Cai et al. 2023; Lo et al. 2024). To study partridgeberry floral VOCs as the flower aged, the production of the monoterpenes α‐pinene and D‐limonene (Figure 3) was measured in flowers 1–5 DAF. Of the four prevalent compounds identified in the VOC profile (Table 2), α‐pinene and D‐limonene were selected because they were quantifiable using the room temperature extraction while camphene and verbenone were not. Because it was determined statistically that there was no difference between the pin and thrum VOC profiles (Table 3), flowers were grouped according to DAF only. Statistical analysis confirms that α‐pinene (Figure 3A) had a significant correlation with DAF (*t* = −4.727 and *p* < 0.0001), D‐limonene concentrations (Figure 3B) were also related with DAF (*t* = −3.282 and *p* = 0.001). As can be seen in Figure 3, for both compounds, there is significant variation in each DAF. This is possibly due to the state of the flower's fertilization (Marshall et al. 2010; Burdon et al. 2015) or other confounding variables such as temperature or humidity at the time of flower collection (Farré‐Armengol et al. 2020).

## 5 Conclusion

To our knowledge, this is the first study that has identified the major VOCs produced by *Mitchella repens* , demonstrating that there is no statistical difference between the VOC profiles of the two floral morphs. Two dominant monoterpenes, α‐pinene and D‐limonene, were followed as flowers aged. Both compounds statistically decreased with floral age. In addition, insect visits to the flowers were observed to decrease with floral age, following the decline of α‐pinene and D‐limonene.

## Conflicts of Interest

The authors declare no conflicts of interest.

## Institutional Review Board Statement

No human subjects nor animals were used in this study.

## Supporting information


Data S1.

## Data Availability

The data used in this study are freely available as supplemental data and can be accessed at https://drive.google.com/drive/folders/1iug4x9jvRJSR5Q_PufttPqA1YFmYWy6d?usp=drive_link.
